# Comparative differential proteomic analysis of minimal change disease and focal segmental glomerulosclerosis

**DOI:** 10.1186/s12882-017-0452-6

**Published:** 2017-02-03

**Authors:** Vanessa Pérez, Dolores López, Ester Boixadera, Meritxell Ibernón, Anna Espinal, Josep Bonet, Ramón Romero

**Affiliations:** 1 Laboratory of Experimental Nephrology, Institut d’Investigació en Ciències de la Salut Germans Trias i Pujol, Universitat Autònoma de Barcelona, Badalona, Spain; 2 Department of Nephrology, Hospital Universitari Germans Trias i Pujol, Universitat Autònoma de Barcelona, Carretera del Canyet s/n, ES-08916 Badalona, Barcelona Spain; 3 Department of Pathology, Hospital Universitari Germans Trias i Pujol, Universitat Autònoma de Barcelona, Badalona, Spain; 4 Applied Statistics Service, Universitat Autònoma de Barcelona, Bellaterra, Spain; 5 Department of Medicine, Universitat Autònoma de Barcelona, Badalona, Spain

**Keywords:** Focal segmental glomerulosclerosis, Glomerular disease, Mass spectrometry, Minimal change disease, Proteomics, Urine, 2D-DIGE

## Abstract

**Background:**

Minimal change disease (MCD) and primary focal segmental glomerulosclerosis (FSGS) are glomerular diseases characterized by nephrotic syndrome. Their diagnosis requires a renal biopsy, but it is an invasive procedure with potential complications. In a small biopsy sample, where only normal glomeruli are observed, FSGS cannot be differentiated from MCD. The correct diagnosis is crucial to an effective treatment, as MCD is normally responsive to steroid therapy, whereas FSGS is usually resistant.

The purpose of our study was to discover and validate novel early urinary biomarkers capable to differentiate between MCD and FSGS.

**Methods:**

Forty-nine patients biopsy-diagnosed of MCD and primary FSGS were randomly subdivided into a training set (10 MCD, 11 FSGS) and a validation set (14 MCD, 14 FSGS). The urinary proteome of the training set was analyzed by two-dimensional differential gel electrophoresis coupled with mass spectrometry. The proteins identified were quantified by enzyme-linked immunosorbent assay in urine samples from the validation set.

**Results:**

Urinary concentration of alpha-1 antitrypsin, transferrin, histatin-3 and 39S ribosomal protein L17 was decreased and calretinin was increased in FSGS compared to MCD. These proteins were used to build a decision tree capable to predict patient’s pathology.

**Conclusions:**

This preliminary study suggests a group of urinary proteins as possible non-invasive biomarkers with potential value in the differential diagnosis of MCD and FSGS. These biomarkers would reduce the number of misdiagnoses, avoiding unnecessary or inadequate treatments.

**Electronic supplementary material:**

The online version of this article (doi:10.1186/s12882-017-0452-6) contains supplementary material, which is available to authorized users.

## Background

Minimal change disease (MCD) and primary focal segmental glomerulosclerosis (FSGS) are glomerular diseases defined by lesions of the podocyte. These diseases are main causes of idiopathic nephrotic syndrome in children and adults and are characterized by proteinuria, hypoalbuminemia, hyperlipidemia and edema, without an underlying etiology [1, 2]. The final diagnosis of glomerular diseases is based on renal biopsy findings and their correlation with clinical, laboratory and serological results. Moreover, renal biopsy is useful for determining the prognosis and for choosing the most appropriate treatment, although the invasiveness of this technique may lead to serious complications [3–5].

Anatomopathologic study combines conventional light microscopy, immunohistology and electron microscopy, and requires an adequate amount of tissue, with a sufficient number of glomeruli to evidence the lesion [6–8].

Light microscopy shows normal glomeruli in MCD and segmental scarring in some, but not all, glomeruli in FSGS. In both entities, electron microscopy typically demonstrates specific ultrastructural findings of diffuse effacement of podocytes’ foot processes in the absence of electron-dense deposits [9, 10]. Due to the focal nature of FSGS, it is complicated to identify this lesion if no affected glomeruli are sampled in the biopsy, and a misdiagnosis of these patients as MCD may occur [8]. The correct diagnosis is crucial to an effective treatment, as MCD is typically responsive to steroid therapy with excellent long-term prognosis, whereas FSGS is usually resistant to steroid therapy and has progressive glomerular filtration rate loss [11, 12]. Consequently, the different therapy approach and the toxicity of steroids make it especially important to differentiate between these disorders.

During the last decades, major technological advances in the field of proteomics have greatly encouraged the search for diagnostic biomarkers of diseases in biological fluids, because extracellular proteins provide valuable information on the physiological state of the entire organism and of specific organs. For this purpose, two-dimensional gel electrophoresis coupled with mass spectrometry (MS) is a commonly used approach. Recently, two-dimensional differential gel electrophoresis (2D-DIGE) has emerged, in which various protein sources are fluorescently labeled, mixed, and run simultaneously on the same polyacrylamide gel. This methodology allow the separation and quantitative analysis of two or more different protein samples within the same gel, reducing gel to gel variation and overcoming the reproducibility and sensitivity limitations of the traditional two-dimensional gel electrophoresis [13].

Among the different biological fluids, urine has the advantage of being obtained easily and non-invasively, in large amounts, and at minimum cost. In addition, urine contains proteins from plasma and from the kidneys, reflecting both systemic and renal physiology. Several studies have been conducted to identify urinary biomarkers of kidney diseases [14–18].

In this study, the urinary proteome of a group of MCD and FSGS biopsy-diagnosed patients was compared aiming to find out candidate biomarkers capable to differentiate between these glomerular diseases.

## Methods

### Patients

In the period between January 2007 and December 2013, 49 patients biopsy-diagnosed of MCD (*n =* 24) and primary FSGS (*n =* 25) were included in this prospective study. Inclusion criteria were: i) Caucasian race, ii) >18 years old, iii) diagnosis achieved by renal biopsy during the initial nephrotic syndrome presentation and before starting any pharmacological therapy (steroids, immunosuppressant drugs, angiotensin converting enzyme inhibitors, angiotensin receptor blockers, etc.), iv) stable renal function (follow-up two years after diagnosis). Clinical or pathological features indicating a secondary cause such as autoimmune diseases, infections, cancer or exposure to nephrotoxic drugs were excluded.

Urine and blood samples were collected the same day of renal biopsy, prior to performing it. All samples were processed identically.

The Research Ethics Committee of the Germans Trias i Pujol Hospital approved the study protocol and all patients gave their written informed consent to participate.

### Study design

MCD and FSGS patients were randomly subdivided into a training set (10 MCD, 11 FSGS) used to perform the 2D-DIGE analysis, and a validation set (14 MCD, 14 FSGS) used to validate the results.

### Renal biopsy

Patients’ histological diagnosis was achieved by a percutaneous renal biopsy.

Biopsies were performed using a Bard Monopty Disposable Core Biopsy Instrument (Bard Biopsy Systems, Tempe, AZ, USA) under ultrasound guidance and routinely processed for light microscopy, immunofluorescence, and electron microscopy examination according to established protocols and image analysis techniques. Light microscopy sections were stained hematoxylin and eosin, periodic acid Schiff, silver methenamine, Masson’s trichrome and Congo red. Immunofluorescence was performed by incubating cryostat sections with polyclonal fluorescein isothiocyanate-conjugated secondary antibodies against IgG, IgM, IgA, C3, C1q, C4, kappa, lambda and fibrinogen (Dako, Glostrup, Denmark). Tissue samples for electron microscopy were processed according to established techniques. Briefly, samples were fixed in 2% glutaraldehyde in phosphate buffer, post-fixed in 1% osmium tetroxide and embedded in epon epoxy resin. Ultrathin sections were stained with uranyl acetate and lead citrate.

### Anthropometric and biochemical parameters

Body surface area was calculated according to Dubois method [19]. Serum creatinine levels were determined using a modified Jaffe kinetic reaction (Roche Diagnostics, Basel, Switzerland). All patients underwent a complete haematological study that included serum glucose (hexokinase method) and serum protein (biuret method). Twenty-four hour proteinuria was measured spectrophotometrically on a Cobas u711 analyzer (Roche Diagnostics) according to the manufacturer’s instructions.

### Urine collection

A first morning void was collected from all patients into a sterile plastic tube and immediately centrifuged at 2,100 g for 30 min at 4 °C to remove cell debris and particulate matter. The supernatant was recovered, adjusted to neutral pH with 1 M NH_4_HCO_3_, aliquoted, and immediately frozen at −80 °C until further analysis.

### Sample labeling and two-dimensional gel electrophoresis

The subset of samples from the training set were pooled together (10 MCD in sample #1 and 11 FSGS in sample #2), adding an equal amount of protein from each patient (500 μg). Total protein concentration was assessed with the Quick Start Bradford protein assay kit (Bio-Rad Laboratories, Hercules, CA, USA) according to manufacturer instructions.

Pooled samples were centrifuged at 10,000 g for 10 min and the supernatant was precipitated by 2DE-CleanUp (GE Helthcare Life Science, Piscataway, NJ, USA). The pellets were resuspended in 100 μl of lysis buffer (8 M Urea, 2.5% CHAPS, 2% ASB-14 and 30 mM Tris–HCl, pH 8.5).

To compare the urine proteomes of both glomerular entities, 75 μg of sample #1 and 75 μg of sample #2 were labeled with different CyDye fluorofors (Cy2 for a pool of both samples, Cy3 for sample #1 and Cy5 for sample #2) before the two-dimensional polyacrylamide gel electrophoresis (2D-PAGE). Each sample was labeled with 8 pmol of CyDye per μg of protein and incubated on ice for 30 min in the dark. The labelling reaction was quenched by adding 1 μl of 10 mM lysine and incubated on ice for 10 min in the dark, according to manufacturer’s instructions (GE Healthcare Life Science).

2D-PAGE with immobilized pH gradient was carried out according to Görg et al. [20]. The labelled samples #1 and #2 were mixed together and then run in the first-dimension by isoelectric focusing (IEF), using the cup-loading method, onto previously rehydrated 24 cm IPG drystrips (GE Healthcare Life Science) with immobilized linear 3–10 pH gradient. IEF was performed at 300 V for 1 h, followed by 3 gradient steps (1000 V for 30 min, 5000 V for 80 min, and 8000 V for 30 min) and finally 8000 V for 2 h. On completion of the IEF, the strips were equilibrated and proteins separated on the second-dimension on a 12% polyacrylamide gel. The electrophoresis was performed at 14 °C until the front of fast migrating ions reached the bottom of the gel. The analytical gels were run in triplicate.

Fluorescence images of the gels were acquired on a Typhoon 9400 scanner (GE Healthcare Life Science) at appropriate wavelengths for Cy3 and Cy5 dyes, and at a resolution of 100 μm. Digitalized images were evaluated using SameSpots v4.0 software (TotalLab Ltd., Newcastle, UK).

### Spot picking and mass spectrometric protein identification

Preparatory 2D-PAGE gels were run to be visualized by colloidal Coomassie staining. Stained gels were scanned with Typhoon scanner and resulting images were matched and aligned with the previous Cy3 and Cy5 fluorescence images. Those spots whose protein abundance was increased or decreased more than 1.5-fold were listed for being identified by matrix-assisted laser desorption/ionization time of flight (MALDI-TOF) peptide mass fingerprinting. The spots of interest were excised from the polyacrylamide gel, destained, and digested with 30 ng of sequencing grade trypsin (Promega, Madison, WI, USA) for 4 h at 37 °C. Peptides were eluted by centrifugation with 40 μl of acetonitrile:H_2_O (1:1) and 0.2% trifluoroacetic acid.

For MS analysis, the samples were prepared by mixing 0.5 μl of sample with the same volume of a solution of alpha-cyano-4-hydroxycinnamic acid matrix (10 mg/ml in 30% acetonitrile, 60% water, and 0.1% trifluoroacetic acid) and were spotted onto a ground steel plate (Bruker Daltonics, Bremen, Germany) and allowed to air-dry. MS spectra were recorded in the positive ion mode on an ultrafleXtreme time-of-flight instrument (Bruker Daltonics). Ion acceleration was set to 25 kV. All mass spectra were externally calibrated using a standard peptide mixture (Bruker Daltonics).

Protein identifications were carried out by Mascot search engine (Matrix Science, Boston, MA, USA), against the NCBInr protein database with the following parameters: 3 maximum missed trypsin cleavages, cysteine carbamidomethylation and methionine oxidation as variable modifications and 50 ppm tolerance.

### Enzyme-linked Immunosorbent assay (ELISA)

The concentration of the proteins identified was assessed using commercially available ELISA kits (Additional file 1) according to manufacturer’s instructions. Each sample was assayed in duplicate. Absorbance optical density values were read fluorometrically at 450 nm on a Varioskan Flash spectral scanning reader (Thermo Fisher Scientific, Vantaa, Finland). The measured concentrations were assessed with the SkanIt Software for Varioskan Flash (version 2.4.1) by extrapolation from a standard curve generated from the standards supplied in the kits.

### Statistical analyses

The first step was performed using univariate and bivariate analyses. For continuous variables, expressed as median (interquartile range), groups were compared using the non-parametric Kruskal-Wallis test. For categorical variables, differences among groups were tested using Likelihood Ratio Chi-Square statistic.

A decision tree [21] was performed to obtain the set of the most discriminative proteins between MCD and FSGS patients. Ten 5-fold cross-validations were performed aiming to validate the decision tree. In addition, for the validation of the decision tree analysis, the corresponding area under the ROC curve (AUC) was calculated.

Statistical analyses were performed with the SAS software v9.3 (SAS Institute Inc., Cary, NC, USA). Significance level was fixed at 0.05.

## Results

Demographical and clinical data of patients are presented in Table 1.

**Table 1:** Demographic and clinical characteristics of the study population

	Training Set			Validation Set			*P*_T-V_	
	MCD	FSGS	*P*	MCD	FSGS	*P*	MCD	FSGS
No. of subjects	10	11		14	14			
Age (years)	39.5 (28.0 –68.0)	57.0 (31.0 –71.0)	0.57	55.5 (30.0 –72.0)	54.5 (36.0 –59.0)	0.45	0.32	0.85
Female/male ratio	3/7	5/6	0.47	6/8	2/12	0.09	0.52	0.08
Body mass index (kg/m^2^)	27.3 (21.9 –33.8)	25.5 (24.0 –26.6)	0.68	29.4 (24.2 –30.6)	26.3 (25.1 –28.4)	0.63	0.76	0.41
Body surface area	1.7 (1.7 –1. 9)	1.8 (1.6 –1.8)	0.83	1.8 (1.6 –1.9)	1.9 (1.8 –2.0)	0.35	0.52	0.11
Serum glucose (mg/dl)	91 (81–101)	88 (83–97)	0.89	84 (81–94)	94 (87–97)	0.19	0.44	0.67
Serum protein (g/dl)	4.4 (3.7 –4.7)	5.0 (4.1 –6.2)	0.09	4.7 (4.1 –4.9)	6.1 (4.5 –6.3)	0.05	0.29	0.56
Serum creatinine (mg/dl)	0.9 (0.8 –1.0)	1.2 (0.9 –1.2)	0.12	0.9 (0.8 –1.3)	1.3 (0.9 –1.8)	0.24	0.64	0.62
Proteinuria (g/24 h)	10.6 (2.3 –12.2)	3.5 (2.5 –7.4)	0.29	9.7 (5.9 –15.0)	3.4 (1.7 –4.5)	0.004	0.52	0.64

Data are shown as median (interquartile range)

Differences between groups were tested using the non-parametric Kruskall Wallis test. *P*_T-V_ shows *P* value between training and validation set. *P* < 0.05 was considered significant

Renal biopsies contained 22.36 ± 11.50 glomeruli.

### 2D-DIGE MS

A total of 394 matched protein spots were detected in 2D-DIGE images (Additional file 2). A total of 242 spots showed a differential abundance when comparing MCD and FSGS (ANOVA, *P* < 0.05); 57.4% and 42.6% were up-regulated in MCD and FSGS, respectively.

Differentially abundant protein spots (with average-fold change > 2 and *P* < 0.01) were targeted for MS analysis. The protein identification gave a total of 25 confident identifications, representing 16 proteins. Eleven of these proteins were up-regulated in MCD patients and 5 were up-regulated in FSGS patients. Table 2 shows the list of the identified proteins; in cases where multiple identifications were made from the same spot, all proteins are reported.

**Table 2:** List of proteins identified in urine from training set using peptide mass fingerprinting

# Spot^a^	*P-value*	MCD	FSGS	Fold^b^	Trend^c^	Protein name	Gene name	UniProt accession no.^d^	Seq. Cov. (%)	Matched peptides	MASCOT Score
1,064	0.004	0.39	1.67	4.3	Down	Branched-chain-amino-acid aminotransferase, mithocondrial	BCAT2	O15382	19.9	5	42.4
1,070	0.004	0.18	2.04	11.5	Down	Nuclear inhibitor of protein phosphatase I	PPP1R8	Q12972	28.2	7	48.4
1,334	<0.001	1.79	0.44	4.1	Up	Alpha-1-antitrypsin	SERPINA1	P01009	21.3	6	44.6
						Platelet-activating factor receptor	PTAFR	P25105	17.3	5	46.5
						Cyclin-Y	CCNY	Q8ND76	32.6	7	49.8
1,352	<0.001	1.79	0.43	4.2	Up	Alpha-1-antitrypsin	SERPINA1	P01009	49.3	13	51.7
1,354	<0.001	1.77	0.40	4.5	Up	Alpha-1-antitrypsin	SERPINA1	P01009	49.3	18	86.3
						Transmembrane channel-like protein 1	TMC1	Q8TDI8	23.9	15	33.4
1,356	<0.001	1.85	0.38	4.8	Up	Transcription elongation factor 1 homolog	ELOF1	P60002	51.8	7	36.2
1,363	<0.001	1.51	0.59	2.6	Up	Transferrin	TF	P02787	24.5	12	64.9
1,392	0.01	0.31	1.57	5.1	Down	Serum albumin	ALB	P02768	28.1	16	58.6
						Leucine-rich repeat-containing protein C10orf11	c10orf11	Q9H2I8	36.9	6	41.6
1,400	0.005	2.18	0.38	5.7	Up	Serum albumin	ALB	P02768	39.4	21	85.1
1,408	0.006	2.17	0.49	4.4	Up	Serum albumin	ALB	P02768	53.2	31	92.1
1,458	0.002	0.28	1.69	6.1	Down	PEST proteolytic signal-containing nuclear protein	PCNP	Q8WW12	51.7	5	38.6
						Branched-chain -amino-acid aminotransferase, mithocondrial	BCAT2	O15382	19.9	5	42.4
1,460	<0.001	0.25	1.72	7	Down	Leucine-rich repeat-containing protein C10orf11	c10orf11	Q9H2I8	36	9	32.7
						Calretinin	CALB2	P22676	32.1	7	27.9
1,468	<0.001	2.22	0.71	3.1	Up	Serum albumin	ALB	P02768	24.8	21	80.2
						39S Ribosomal protein L17, mithocondrial	MRPL17	Q9NRX2	62.9	9	40
						Humanin-like protein 6	HN6	P0CJ73	100	7	64.1
1,469	<0.001	2.98	0.68	4.4	Up	Serum albumin	ALB	P02768	28.2	25	56.6
						Histatin-3	HTN3	P15516	68.6	4	36.7
7,740	<0.001	1.27	0.49	2.6	Up	Zinc-alpha-2-glycoprotein	AZGP1	P25311			155
7,810	<0.001	1.27	0.49	2.6	Up	Zinc-alpha-2-glycoprotein	AZGP1	P25311	28.9	10	61.9

^a^Spot number generated by SameSpots image analysis software, referencing the spots shown on Additional file 2

^b^Ratio of protein expression between MCD and FSGS

^c^Up: up-regulated in MCD compared to FSGS; Down: down-regulated in MCD compared to FSGS

^d^Accession number from NCBInr database

### Validation by ELISA

**Fig. 1:**
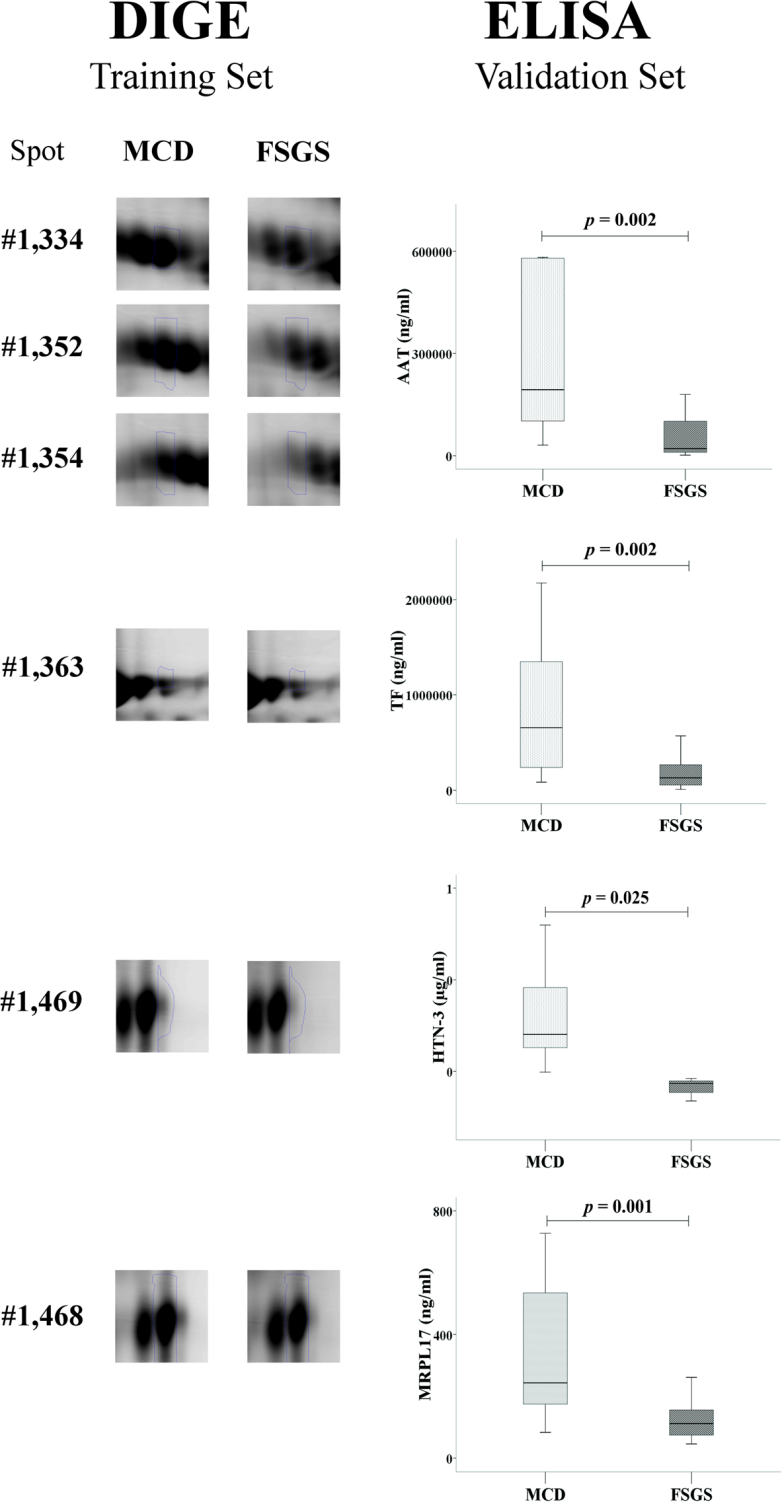
Selection of DIGE spots and validation by ELISA

The results of the validation are shown on Table 3 and Fig. 1.

**Table 3:** Validation results

		N	Urine concentration	*P*
AAT	MCD	13 / 14	193.5 (102.49 –580.0) μg/ml	0.002
	FSGS	14 / 14	20.93 (10.45 –101.65) μg/ml	
TF	MCD	12 / 14	653.63 (241.27 –1,348.38) μg/ml	0.002
	FSGS	14 / 14	129.96 (55.41 –267.10) μg/ml	
HTN-3	MCD	5 / 14	0.35 (0.32 –0.48) μg/ml	0.03
	FSGS	3 / 14	0.22 (0.17 –0.23) μg/ml	
MRPL17	MCD	12 / 14	242.98 (174.25 –534.75) pg/ml	0.001
	FSGS	14 / 14	111.86 (74.90 –154.78) pg/ml	
PCNP	MCD	14 / 14	441.67 (152.50 –503.10) pg/ml	0.72
	FSGS	12 / 14	348.55 (216.25 –437.70) pg/ml	
CALB2	MCD	14 / 14	3.52 (2.88 –4.40) pg/ml	0.002
	FSGS	14 / 14	6.98 (4.29 –8.65) pg/ml	
CCNY	MCD	12 / 14	87.60 (83.40 –91.15) pg/ml	0.71
	FSGS	12 / 14	87.70 (84.30 –102.2) pg/ml	
PTAFR	MCD	5 / 14	0.84 (0.46 –0.98) ng/ml	0.25
	FSGS	2 / 14	0.51 (0.36 –0.66) ng/ml	

N represents the number of urine samples in which the proteins were detected by ELISA

Three DIGE spots, up-regulated in MCD, were identified as alpha-1-antitrypsin (AAT). The concentration of this protein was significantly higher in the urine of MCD patients.

The identification of the DIGE spot #1,334 resulted in 2 proteins, platelet-activating factor receptor (PTAFR) and cyclin-Y, in addition to AAT. By ELISA we found the presence of these proteins in the urine of some patients of the validation set, but no differences were observed when comparing MCD and FSGS.

One DIGE, up-regulated in MCD, was identified as transferrin (TF). By ELISA, we found a higher concentration of this protein in the urine of MCD.

Another spot up-regulated in MCD was identified as Histatin-3 (HTN). This protein was only detected by ELISA in 8 patients, with higher concentration in those diagnosed MCD.

Another spot up-regulated in MCD was identified as 39S ribosomal protein L17, mitochondrial (MRPL17). The concentration of this protein was higher in the urine of MCD patients.

One DIGE up-regulated in FSGS was identified as calretinin (CALB2). This protein was in a higher concentration in urine of FSGS patients.

The spot #1,458, up-regulated in MCD patients, was identified as PEST proteolytic signal-containing nuclear protein. By ELISA, no differences were found.

The rest of proteins identified were not detected by ELISA in the urine of patients from the validation set.

### Decision tree analysis

In the first step for building the decision tree, CALB2 was used for classifying patients. Hence, 2 groups were obtained: 19 patients (14 MCD, 5 FSGS) with levels of CALB2 < 6.4 ng/ml and 9 patients (9 FSGS) with levels of CALB2 > = 6.4 ng/ml. In the second step, for the group who had levels of CALB2 < 6.4 ng/ml, the best partition was using the value of MRPL17 > = 139.29 pg/ml. To conclude, a final partition was defined by the detection of HTN, in the group who had levels of CALB2 < 6.4 ng/ml and MRPL17 < 139.29 pg/ml.

**Fig. 2:**
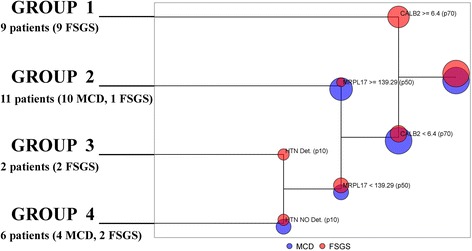
Decision tree analysis

Accordingly, 4 groups of patients were obtained (Fig. 2). Group 1 included 9 patients, all of them FSGS with levels of CALB2 > = 6.4 ng/ml; Group 2 included 11 patients (10 MCD, 1 FSGS) with levels of CALB2 < 6.4 ng/ml and MRPL17 > = 139.29 pg/ml. Moreover, these patients showed high levels of AAT, TF, HTN and PTAFR; Group 3 included 2 FSGS patients with levels of CALB2 < 6.4 ng/ml, levels of MRPL17 < 139.29 pg/ml and detection of HTN; Group 4 included 6 patients (4 MCD, 2 FSGS) with levels of CALB2 < 6.4 ng/ml, levels of MRPL17 < 139.29 pg/ml and no detection of HTN. Groups 2 and 4 were mainly composed of MCD patients, and Groups 1 and 3 included only FSGS patients. Therefore, the predicted-MCD patients were those in Groups 2 and 4, and the predicted-FSGS patients were those in Groups 1 and 3. All MCD patients were classified as predicted-MCD, while 78.6% of FSGS were correctly predicted.

From the validation analysis, the AUC was 0.89 and 95% Confidence Interval = [0.78, 1].

## Discussion

We included a highly selected group of patients with clinical and histological diagnosis of MCD and FSGS. The diagnosis of FSGS is established by the finding of at least a single abnormal glomerulus and it has been stated that the probability of misdiagnosis is statistically relevant when fewer than eight glomeruli are found in biopsy samples [8]. In our study, all tissue samples contained more than eight glomeruli. Moreover, we can state that all patients were correctly classified, as those diagnosed MCD achieved a complete remission, without any relapse for at least two years.

In recent years, several research groups have proposed different urinary biomarkers to differentiate between these glomerular diseases, such as CD80 and TGFβ [22, 23], but there is not enough evidence to use them in clinical practice. These candidate biomarkers need further validation and in a larger cohort.

Of our results, we consider highly interesting the finding of a set of proteins whose concentration in urine was different between these glomerular diseases. With some of these proteins, named calretinin, histatin-3 and 39S ribosomal protein L17, we built a decision tree capable to predict patient’s pathology.

These results were obtained after conducting a proteomic study. Beside direct analysis of renal tissue, urinary proteome study has potential value in the none-invasive diagnosis of kidney diseases diagnosis. We focused on MCD and FSGS in which the histological study may be similar and lead to an erroneous diagnosis. Consequently, our results may be useful to clinicians to confirm the diagnosis and thereby avoid unnecessary or inadequate treatments.

The comparison of the urinary proteome of MCD and FSGS patients was achieved by 2D-DIGE, resulting in 16 proteins as possible biomarkers. Various proteins were identified in different spots, and numerous spots contained more than one protein, making it difficult to attribute abundance changes to a specific protein. For that reason, the results obtained by 2D-DIGE were validated by independent ELISA analyses.

Various DIGE spots that were up-regulated in MCD were identified as AAT. By ELISA we corroborated that this protein was in a higher concentration in the urine of MCD patients. AAT is a 52-kDa glycoprotein and the most abundant circulating serine protease inhibitor of a broad range of proteases, mainly against neutrophil elastase. AAT protects tissues from enzymes released from cells when they are injured and inflamed. Other functions of AAT have been suggested, such as modulating immunity, inflammation and apoptosis [24, 25]. AAT is mainly synthesized in the liver and to a lesser extent by a variety of extra-hepatic tissues, such as renal tubular epithelial cells. Several studies have revealed that AAT protects the kidney by anti-apoptotic and anti-inflammatory routes in renal ischemic/reperfusion injury and it has been proposed as a biomarker for acute kidney injury (AKI) [26–28]. Since AKI can be due to a glomerular injury, we paid attention at the renal function of our patients and observed that there were no differences in serum creatinine levels between MCD and FSGS.

Other studies have described a high presence of AAT in the urine of patients with nephrotic syndrome, and a practical absence in the urine of healthy subjects [29, 30]. In agreement, in a previous study, by analyzing the urinary peptidome, we found one peptide, identified as AAT, that showed a higher intensity in MCD compared with FSGS [31].

The present study also revealed higher levels of TF in MCD. Urinary TF results from abnormal permeability of the glomerular basement membrane, and it has been suggested to be a marker for early stages of glomerular diseases. Increased urinary TF excretion has been suggested to precede the development of microalbuminuria in glomerular diseases [32]. Other studies have found that urinary TF may predict the severity of mesangial cellularity and glomerulosclerosis in the early stages of glomerular diseases [33].

MRPL17 is a protein encoded by nuclear genes and helps in protein synthesis within the mitochondrion. To our knowledge, there are no studies relating this protein with kidney diseases.

We identified CALB2 as another possible candidate biomarker capable of differentiating MCD from FSGS. CALB2, a 29 kDa calcium-binding protein belonging to the troponin C superfamily, is predominantly expressed in specific neurons of the central and peripheral nervous system. This protein is involved in diverse cellular functions including intracellular calcium buffering, messenger targeting, and the modulation of neuronal excitability. CALB2 has been proposed as a diagnostic marker for some human diseases, including Hirschsprung disease and some cancers, such as mesothelioma and lung tumours [34–36].

## Conclusions

In conclusion, given the difficulty in differentiating, in some cases, between MCD and FSGS by evaluation of renal biopsies, it becomes necessary to search for diagnostic biomarkers. In this study, we built a decision tree which seems a good tool for predicting patient’s pathology when there are doubts if it is MCD or FSGS, although future efforts must be made to include more patients and to evaluate its effectiveness.

## Additional files

Additional file 1: (Table) List of commercially available ELISA kits used to validate proteins identified by peptide mass fingerprinting. (PDF 7 kb)
Additional file 2: (Figure) Image of a preparatory 2D-PAGE gel used to pick spots. (JPG 1318 kb)

## Abbreviations

2D-DIGE: Two-dimensional differential gel electrophoresis
2D-PAGE: Two-dimensional polyacrylamide gel electrophoresis
AAT: Alpha-1-antitrypsin
AKI: Acute kidney injury
AUC: Area under the ROC curve
CALB2: Calretinin
ELISA: Enzyme-linked immunosorbent assay
FSGS: Focal segmental glomerulosclerosis
HTN: Histatin-3
IEF: Isoelectric focusing
MALDI-TOF: Matrix-assisted laser desorption/ionization time of flight
MCD: minimal change disease
MRPL17: 39S ribosomal protein L17, mitochondrial
MS: Mass spectrometry
PTAF: Platelet-activating factor receptor
TF: Transferrin
