# Supplementary material for: Comparative differential proteomic analysis of minimal change disease and focal segmental glomerulosclerosis
Source: BMC Nephrol. 2017 Feb 3;18:49. doi: 10.1186/s12882-017-0452-6 (PMC5291957; doi:10.1186/s12882-017-0452-6)
Supplement: Additional file 1: — (Table) List of commercially available ELISA kits used to validate proteins identified by peptide mass fingerprinting. (PDF 7 kb) [file 12882_2017_452_MOESM1_ESM.pdf]

Supplemental Table 1. List of commercially available ELISA kits used to validate proteins identified by peptide mass fingerprinting.

Protein Name	Catalog Number	Type	Dilution	LDL (ng/ml)	Range (ng/ml)	Intra-Assay CV (%)	Inter-Assay CV (%)
Alpha-1 antitrypsin	KA0459[a]	Q	1:5,000 - 1:40,000	0.3	0.39 - 100	4.9	7.0
Transferrin	KA0510[a]	Q	1:10,000 - 1:30,000	1.5	1.56 - 100	4.5	7.1
Zinc-alpha-2-glycoprotein	KA1689[a]	C	1 : 1	0.02	0.1 - 1000	< 10	< 15
Calretinin	E-EL-H0628[b]	Q	1 : 1	0.19	0.31 - 20	< 10	< 10
Transmembrane channel-like protein 1	CSB-EL023633HU[c]	Q	1 : 1	0.006	0.03 - 1.6	< 8	< 10
Histatin-3	CSB-EL010879HU[c]	Q	1 : 1	39	156 - 10,000	< 8	< 10
Nuclear inhibitor of protein phosphatase I	CSB-EL018555HU[c]	Q	1 : 1	0.008	0.003 - 2	< 8	< 10
Leucine-rich repeat-containing protein c10orf11	CSB-EL002909HU[c]	Q	1 : 1	0.005	0.02 - 1.2	< 8	< 10
Transcription elongation factor 1 homolog	CSB-EL007618HU[c]	Q	1 : 1	0.004	0.02 - 1	< 8	< 10
PEST proteolytic signal-containing nuclear protein	CSB-EL017625HU[c]	Q	1 : 1	0.006	0.02 - 1.5	< 8	< 10
Branched-chain-amino-acid aminotransferase, mithocondrial	CSB-EL002601HU[c]	Q	1 : 1	0.006	0.02 - 1.5	< 8	< 10
39S ribosomal protein L17, mitochondrial	CSB-EL014824HU[c]	Q	1 : 1	0.006	0.03 - 1.6	< 8	< 10
Platelet activating factor receptor	SEC753Hu[d]	Q	1 : 1	0.06	0.16 - 10	< 10	< 12
Cyclin Y	SED270Hu[d]	Q	1 : 1	0.03	0.08 - 5	< 10	< 12
Humanin-like protein 6	E12591h[e]	C	1 : 1		0.31 - 20		

LDL is the sensitivity, the Lowest Detectable Limit, defined as the lowest protein concentration that could be differentiated from zero. Q means Quantitative Sandwich ELISA and C means Competitive ELISA.

[a] Abnova (Heidelberg, Germany); [b] Elabscience (Wuhan, China); [c] Cusabio (Wuhan, China); [d] Cloud-Clone Corp. (Houston, TX, USA); [e] Wuhan EIABB Science (Wuhan, China)
