# Supplementary figures and images for: Comparative differential proteomic analysis of minimal change disease and focal segmental glomerulosclerosis
Source: BMC Nephrol. 2017 Feb 3;18:49. doi: 10.1186/s12882-017-0452-6 (PMC5291957; doi:10.1186/s12882-017-0452-6)

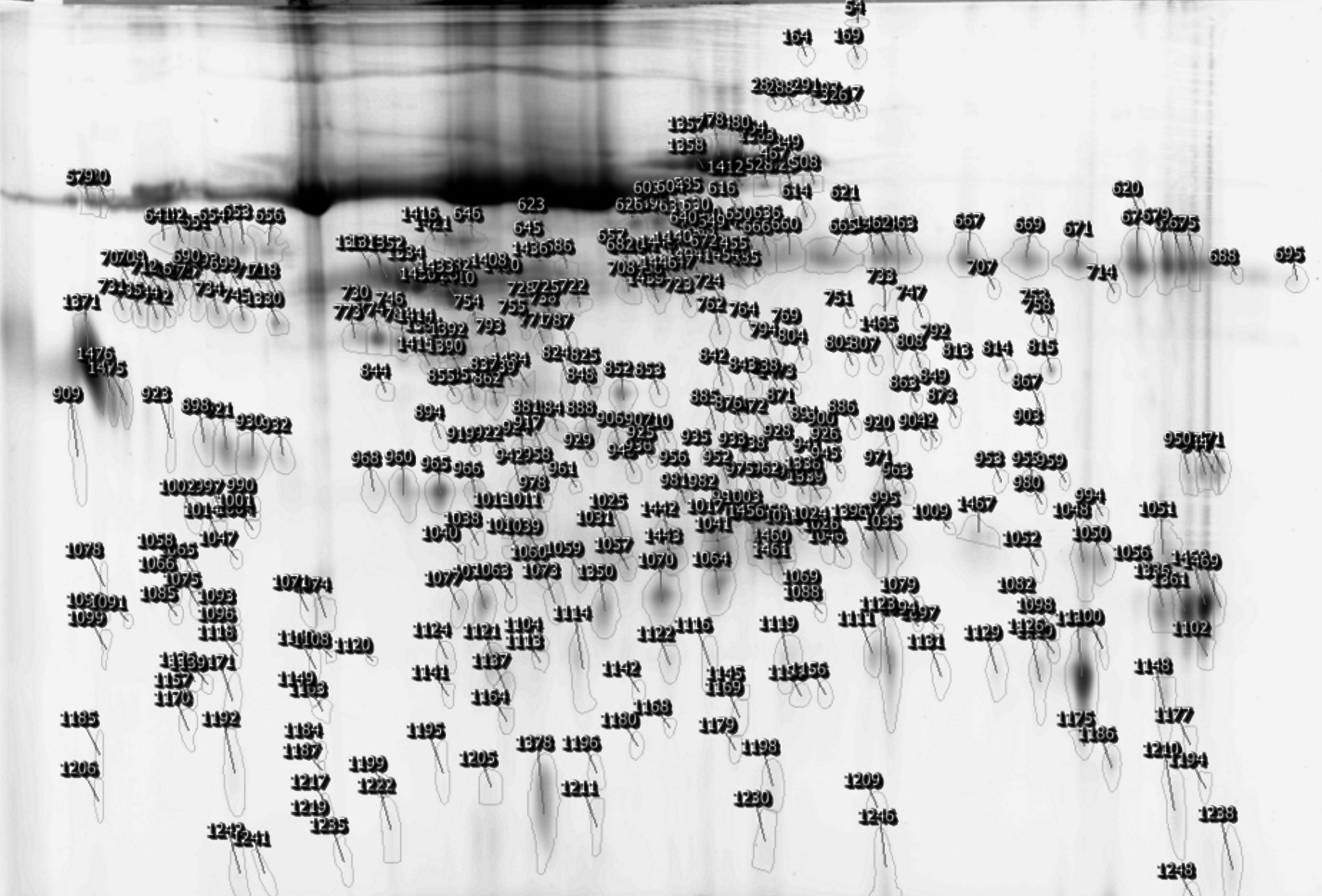

Supplement: Additional file 2: — (Figure) Image of a preparatory 2D-PAGE gel used to pick spots. (JPG 1318 kb) [file 12882_2017_452_MOESM2_ESM.jpg]
